# ST6GAL1 and α2-6 Sialylation Regulates IL-6 Expression and Secretion in Chronic Obstructive Pulmonary Disease

**DOI:** 10.3389/fimmu.2021.693149

**Published:** 2021-07-05

**Authors:** Stefanie Krick, E. Scott Helton, Molly Easter, Seth Bollenbecker, Rebecca Denson, Rennan Zaharias, Phillip Cochran, Shia Vang, Elex Harris, James M. Wells, Jarrod W. Barnes

**Affiliations:** ^1^ Division of Pulmonary, Allergy and Critical Care Medicine, Department of Medicine, The University of Alabama at Birmingham, Birmingham, AL, United States; ^2^ Gregory Fleming James Cystic Fibrosis Research Center, The University of Alabama at Birmingham, Birmingham, AL, United States; ^3^ UAB Lung Health Center, Birmingham, AL, United States; ^4^ Birmingham VA Medical Center, Birmingham, AL, United States

**Keywords:** ST6GAL1, COPD, cigarette smoke, bronchial epithelium, inflammation

## Abstract

Chronic obstructive pulmonary disease (COPD) is a systemic disease strongly associated with cigarette smoking, airway inflammation, and acute disease exacerbations. Changes in terminal sialylation and fucosylation of asparagine (N)-linked glycans have been documented in COPD, but the role that glycosyltransferases may play in the regulation of N-linked glycans in COPD has not been fully elucidated. Recent studies suggest that modulation of ST6GAL1 (ST6 beta-galactoside alpha-2,6-sialyltransferase-1), which catalyzes terminal α2-6 sialylation of cellular proteins, may regulate inflammation and contribute to COPD phenotype(s). Interestingly, it has been previously demonstrated that ST6GAL1, a Golgi resident protein, can be proteolytically processed by BACE1 (beta-site amyloid precursor protein cleaving enzyme-1) to a circulating form that retains activity. In this study, we showed that loss of ST6GAL1 expression increased interleukin (IL)-6 expression and secretion in human bronchial epithelial cells (HBECs). Furthermore, exposure to cigarette smoke medium/extract (CSE) or BACE1 inhibition resulted in decreased ST6GAL1 secretion, reduced α2-6 sialylation, and increased IL-6 production in HBECs. Analysis of plasma ST6GAL1 levels in a small COPD patient cohort demonstrated an inverse association with prospective acute exacerbations of COPD (AECOPD), while IL-6 was positively associated. Altogether, these results suggest that reduced ST6GAL1 and α2-6 sialylation augments IL-6 expression/secretion in HBECs and is associated with poor clinical outcomes in COPD.

## Introduction

The glycosylation of proteins and lipids has been shown to be critically involved in the regulation of a variety of physiological and pathological processes in eukaryotic cells (1–3). ST β-galactoside alpha-2,6-sialyltransferase 1 (ST6GAL1) is a type II membrane protein that is commonly localized in the Golgi apparatus catalyzing the transfer of a sialic acid from Cytidine 5′-monophosphate (CMP)-sialic acid to galactose-containing glycans (4). ST6GAL1 plays an important role in cancer progression and metastasis (5–8). The expression of ST6GAL1 has been determined to be downregulated in some cancers including bladder cancer and upregulated in others such as prostate, lung, and breast cancer (9–11). Interestingly, ST6GAL1 has been shown to regulate Notch1, Hes1, matrix metalloproteinases (MMPs) and vascular endothelial growth factor (VEGF) in lung cancer and altered α2-6 sialylation has been linked to lung cancer progression (10). ST6GAL1 has also been documented for its role in other cancer cellular processes including angiogenesis (12, 13), inflammation (14, 15), and apoptotic resistance (16–18).

In the lung, it has been shown that ST6GAL1 mRNA levels were significantly increased in non-small cell lung cancer, whereas other sialyltransferases were downregulated, such as ST3GAL1, ST6GALNAC3, and ST8SIA6 (10). In addition, α2,6-sialylation by ST6GAL1 has been linked to lung cancer progression by mediating tumor invasiveness and protecting cancer cells through hypoxia inducible factor (HIF)-1α signaling (19). Recently, ST6GAL1 was linked to modulating airway mucins and sialylation levels in asthma, which further altered cell proliferation and inflammation in this disease (20). The literature on ST6GAL1 in other chronic lung diseases; however, is limited and its role in underlying lung disease processes has not been fully elucidated.

Chronic obstructive pulmonary disease (COPD) is the third leading cause of death globally and is strongly associated with cigarette smoke and airway inflammation with disease exacerbations being a prognostic factor increasing the mortality of the disease (21–23). Inflammation has been shown to lead to alterations in protein glycosylation (24–27) and assessment of plasma from individuals with COPD demonstrated significant changes in compound glycan structures such as tetra-sialylated and complex-type fucosylated glycoforms (28). In addition, alterations in asparagine (N)-linked glycans have been documented for their role in COPD (29) and the function of α1-antitrypsin (30, 31). Still, studies investigating the role of terminal glycosylation and the potential role of glycosyltransferases in the regulation and function of the N-linked glycans in COPD have not been fully elucidated.

In this study, our goal was to determine the effects of gain- and loss-of-function of ST6GAL1 on the response of human bronchial epithelial cells (HBECs) to inflammatory stimuli, and identify the clinical relevance for changes in circulating ST6GAL1 in smoking, COPD, and acute exacerbations of COPD (AECOPD). Our findings show that loss of ST6GAL1 and α2-6 sialylation increases interleukin (IL)-6 expression/secretion in HBECs similar to cigarette smoke and ST6GAL1 cleavage/secretion blockade. In addition, reduced circulating ST6GAL1, while increased IL-6 levels in the same COPD patient cohort, was shown to associate with prospective AECOPD.

## Materials and Methods

### Study Approval

All protocols were approved by the Institutional Review Board of the University of Alabama at Birmingham and written consent was obtained from each patient enrolled in the study. The research herein was performed in accordance with the Helsinki Declaration.

### Study Population

Individuals with COPD as defined previously (32) were recruited by the University of Alabama at Birmingham (UAB) Lung Health Center from May 2017 through December 2018. These patients were classified based on severity of the COPD using the Global Initiative for Chronic Obstructive Lung Disease (GOLD) guidelines (33), which are as follows: GOLD 1: Mild (FEV1 ≥ 80% predicted); GOLD 2: Moderate (FEV1 between 50 and 79% predicted); GOLD 3: Severe (FEV1 between 30 and 49% predicted); and GOLD 4: Very severe (FEV1 <30% predicted). Subjects were recruited during their stable state and followed prospectively for one year. Data collection included demographic data, smoking history, pre- and post-bronchodilator spirometry using American Thoracic Society (ATS) standards (34), dyspnea assessment using the modified medical research questionnaire (MMRC), respiratory symptom assessment using the Breathlessness, Cough, and Sputum Scale (BCSS), and queried for AECOPD within the previous 12-months prior to the study visit. AECOPD was defined as a persistent worsening of the subject’s condition from a stable state that was acute in onset, lasted more than 48 hours, and required additional treatment (35, 36). Inclusion criteria to participate was to have a diagnosis of COPD (35) and willing to sign the inform consent to participate.

### Blood Sampling and ST6GAL1 Measurements

Venous blood was sampled during the study visit and processed immediately by centrifugation and collection of the plasma fraction with subsequent storage at −80°C. Specimens were thawed within 6 months and ST6GAL1 levels were measured by enzyme-linked immunosorbent assay (ELISA) utilizing the Human ST6GAL1 ELISA (RAB1722; Sigma, St. Louis, MO; USA) as per provided protocol.

### Bronchial Epithelial Cell Cultures

16HBE cells (HBECs), an immortalized human bronchial epithelial cell line, were plated and grown as recently described (37). All treatments were carried out in antibiotic-free Eagle’s Minimum Essential Medium (EMEM; ATCC, Manassas, VA; USA) with 1x GlutaMAX (Gibco; Gaithersburg, MD; USA) and 10% fetal bovine serum (Atlas Biologicals; Fort Collins, CO; USA) on cell culture plates coated with collagen IV. Cigarette-smoked medium/extract (CSE) was prepared by bubbling cigarette smoke through 1.0 ml serum-free EMEM per cigarette, followed by sterile-filtering through a 0.45 µm filter, and subsequent spectrophotometric analysis to define concentration. For experimental purposes, 100% CSE was set at an OD = 1.0 when absorbance was measured at 320 nm. Short-term CSE exposure was performed on HBECs plated at 6.0 x 10^4^ cells per well in 12-well plates. Following overnight incubation to allow for cell attachment, the cells were treated with varied concentrations of CSE for 24 hours. Long-term CSE exposure was performed on HBECs following a continuous culture method utilizing 3-day intervals for media changes between passages. Briefly, cells were collected by trypsinization, plated at 1.5 x 10^5^ cells per well on a 6-well plate, and treated with 2.5 ml medium containing CSE every 3 days. The remaining cells from each well were washed in cold phosphate buffered saline (PBS) and pelleted at 300 x g. Cell pellets and conditioned media for each time point were stored at -80°C until time of analysis.

For β-site amyloid protein cleaving enzyme 1 (BACE1) inhibitor studies, 16HBE cells were plated as described above followed by a 24-hr recovery period. Then, cells were incubated for 2 hours with either DMSO vehicle or 20 μM LY2886721 (Selleck Chem; UK), which is a small molecule inhibitor of BACE1/2; hereafter referred to as iBACE. After pre-incubation with iBACE, CSE was added and cells were incubated for an additional 72 hours.

### Knockdown and Overexpression of ST6Gal in Bronchial Epithelial Cells

HBECs were generated utilizing lentiviral transduction particles containing a non-mammalian shRNA control sequence (pLKO.1-puro SHC002V; Sigma, USA); MISSION shRNA targeted against ST6GAL1 (SHCLNV-NM_003032; Sigma, USA); or an expression cassette for overexpression of ST6GAL1 (M0351; GeneCopoeia; Rockville, MD; USA). Cells were plated at a density of 4.0 x 10^4^ cells per well in 24-well plates and infected overnight with 10 TU per cell in Opti-MEM (Gibco, USA) containing 8 µg/ml Polybrene (Sigma, USA). Medium was changed to EMEM with GlutaMAX and 10% fetal bovine serum and cells were allowed to recover for 24 hours. Following the recovery period, cells were selected with puromycin (10 µg/ml) for 3 days, and then maintained in 0.5 μg/ml puromycin. Knockdown and overexpression were confirmed by Western blot and quantitative RT-PCR.

### RNA Purification and Quantitative RT-PCR

RNA was extracted using the GeneJET RNA purification kit (Thermo Scientific, Grand Island, NY, USA). For gene expression analysis, qRT-PCR was performed using the following Taqman probes (Life technologies/Applied Biosystems; Carlsbad, CA; USA): Hs00949382 for ST6GAL1; Hs01555410_m1 for IL1β; Hs00174131 for IL6; and Hs00174103_m1 for IL8. Hs02758991 for GAPDH was used as our internal control and the transcript expression data for each gene was normalized to GAPDH.

### Flow Cytometry

Sialylation was assessed utilizing FITC-conjugated *Sambucus nigra* agglutinin (SNA)-FITC; Vector Labs; Burlingame, CA; USA), which is a lectin that binds preferentially to α2-6 linked sialic acid as previously described (25). Briefly, HBECs were gently dissociated using Accutase (Gibco, USA), washed with cold Dulbecco’s phosphate buffered saline containing 0.1mM calcium (DPBS; Gibco, USA), and stained for 1 hour in the dark at 4°C with 10 µg SNA-FITC per ml DPBS. Cells were washed twice in DPBS and fixed in 1% paraformaldehyde. Stained cells were analyzed on a LSR II Flow cytometer and the data were analyzed utilizing FlowJo software (BD Life Sciences; Franklin Lakes, New Jersey; USA).

### IL-6 and IL-8 ELISA

IL-6 and IL-8 cytokine levels in cell culture media were measured utilizing a human IL-6 and a human IL-8 enzyme-linked immunosorbent assays (ELISA) from Invitrogen (Thermo Scientific). Briefly, media from 16HBE cell cultures were collected after treatment at indicated time points, clarified by centrifugation at 500 x g for 10 min at 4°C, loaded onto an assay plate coated with anti-IL-6 or anti-IL-8 capture antibody, and incubated for 2 hours at room temperature. After completing the manufacturer’s suggested protocol, absorbance was measured at 450 nm.

### ST6GAL1 Slot Blot

Samples were prepared in sample loading buffer containing SDS and DTT. Equal volumes of samples were transferred onto a 0.45μm nitrocellulose membrane under gentle vacuum using Bio-Rad Bio-Dot SF microfiltration apparatus (Bio-Rad, Life Sciences, USA). To ensure equal loading, the supernatants were normalized to total cellular protein following a Bradford assay and loaded equivalently. Slot blots were probed with a monoclonal ST6GAL1 antibody (MA5-11900; Thermo Scientific; Grand Island, NY; USA). The secondary antibody used was a goat anti-mouse IgM antibody conjugated to HRP. Blots were developed using enhanced chemiluminescence SuperSignal West Dura Substrate (Thermo Scientific) and imaged using the GE Imaging System (GE Healthcare). ImageJ software (38, 39) was used to perform densitometry measurements.

### Statistics

Data were expressed as mean ± SEM, median [interquartile range or IQR], and counts (percentages). Student’s *t* tests were used to analyze group differences for continuous variables; Mann Whitney U tests were used to measure between group differences for ST6GAL1 given its non-Gaussian distribution in the human cohort; and 1-way ANOVA or Kruskal Wallis tests with appropriate post-hoc tests were used to measure between-group differences for analyses that involved three or more groups. Pearson’s correlation coefficients were used to measure correlation between ST6GALl and outcomes for COPD in the clinical cohort. Logistic regression models adjusting for post-BD FEV1 percent predicted smoking status (current *vs* not-current) were used to measure the association between ST6GAL1 and AECOPD at 1-year of follow-up. SPSS (version 26.0, Chicago, IL, USA) and PRISM (Version 9, GraphPad Software, Inc., La Jolla, CA) was used for all statistical analyses. A p-value of less than 0.05 was considered statistically significant.

## Results

### Sialylation Is Reduced by Cigarette Smoke Extract in HBECs

**Figure 1:**
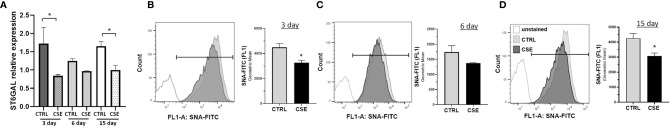
Reduced extracellular sialylation in HBEs following 3, 6 and 15-day CSE exposure. **(A)** Relative mRNA expression of ST6GAL1 at times indicated without (CTRL) and with cigarette smoke extract (CSE). **(B–D)** Flow cytometric histogram showing levels of α2-6 sialylation for HBECs cultured for 3, 6, and 15 days without (CTRL) and with (CSE). Ten thousand events were collected for each group, analyzed by SNA-FITC staining, and shown as the geometric mean of the values. Experiments were performed in triplicate and three separate experiments. SNA, Sambucus Nigra Lectin; and CSE, cigarette smoke extract. All bar graphs are means ± SEM with *p < 0.05.

Previous reports have shown that glycosylation is altered in COPD, and reduced following, cigarette smoke exposure and may result in inflammation (24–28, 40). To determine whether cigarette smoke results in changes in ST6GAL1 expression and/or α2-6 sialylation, HBECs were subjected to mRNA transcript analysis and SNA-FITC staining (which preferentially binds to α2-6 sialic acid on terminal galactose/N-acetylglucosamine over the α-2,3 linkage) and flow cytometric analysis following exposure to CSE for 3, 6, and 15 days. Interestingly, mRNA expression was reduced at 3, 6, and 15 day of CSE, reaching statistical significance at 3 and 15 days (**Figure 1A**). Similarly, the levels of α2-6 sialic acid were reduced in the presence of CSE at all three time points analyzed when compared to vehicle-treated cultures (**Figures 1B–D**). These data suggest that ST6GAL1 expression and α2-6 sialylation are reduced following exposure to cigarette smoke extract in HBECs.

### ST6GAL1 Overexpression and Knockdown Leads to Alterations in Sialylation in HBECs

**Figure 2:**
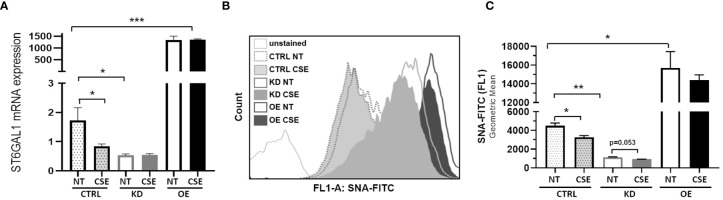
ST6GAL1 overexpression and knockdown leads to alterations in sialylation in HBECs. **(A)** Bar graphs showing ST6GAL1 mRNA levels in stably transfected ST6GAL1 KD and OE cultures compared to control (CRTL) infected cells in the presence or absence of CSE. **(B)** Flow cytometric analysis of α2-6 sialylation using SNA-FITC labeling of HBECs (CTRL, ST6GAL1 KD, and ST6GAL1 OE) following treatment with and without CSE for 3 days. **(C)** Graphical data of the 10,000 events that were collected from **(B)** for each group and analyzed. Abbrv. SNA, Sambucus Nigra Lectin; CTRL, pLKO vector control; KD, ST6GAL1 knockdown; and OE, ST6GAL1 overexpression; NT, no treatment; CSE, cigarette smoke extract; and HBECs, human bronchial epithelial cells. Experiments were performed in triplicate using three separate experiments. All bar graphs are means ± SEM with *p < 0.05, **p < 0.01 and ***p < 0.001.

To determine the consequences of the gain- and loss-of-function of ST6GAL1 in bronchial epithelium, we stably overexpressed or knocked down ST6GAL1 in bronchial epithelial cell cultures. Overexpression showed a marked increase in relative ST6GAL1 mRNA expression, and significant downregulation following siRNA gene knockdown and clonal selection (**Figure 2A**). CSE reduced the levels of ST6GAL1 expression in the control group; however, it did not statistically change the expression levels in the knockdown or overexpression groups (**Figure 2A**). Assessment of α2-6 sialylation in these stably transfected cultures with and without CSE showed a further reduction following ST6GAL1 knockdown (**Figures 2B, C**). As expected, stable overexpression of ST6GAL1 led to an increase in α2-6 sialylation (**Figures 2B, C**). Following 3 days of CSE exposure, the levels of α2-6 sialylation were shown to significantly decrease further when compared to vehicle treatment in the control (**Figures 2B, C**). The mean α2-6 sialylation was slightly reduced in the ST6GAL1 knockdown (p=0.053) and sialylation changes in the overexpressing cell cultures but did not reach statistical significance. These data suggest that overexpression of ST6GAL1 increases α2-6 sialylation and ST6GAL1 knockdown results in similar ST6GAL1 expression and α2-6 sialylation levels as CSE exposure in the HBEC cultures (**Figure 1**).

### ST6GAL1 Knockdown Increases IL-6 Expression and Secretion

**Figure 3:**
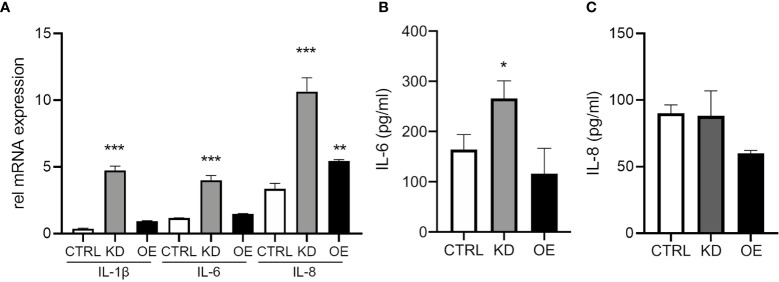
ST6GAL1 knockdown increases IL-6 expression and secretion. **(A)** Relative mRNA transcript levels of IL-1β, IL-6, and IL-8 in CTRL, ST6GAL1 KD, and ST6GAL1 OE HBECs. Analysis of IL-6 **(B)** and IL-8 **(C)** protein levels from supernatants of CTRL, ST6GAL1 KD, and ST6GAL1 OE HBECs using ELISA. CTRL, pLKO vector control; KD, ST6GAL1 knockdown; OE, ST6GAL1overexpression; rel, relative; IL-6, interleukin 6; and IL-8, interleukin 8. All experiments were reproduced 3 times and done in triplicates with bar graphs indicating means ± SEM with *p < 0.05, **p < 0.01 and ***p < 0.001.

Next, we wanted to determine the functional outcomes of altering ST6GAL1 expression on the production of the pro-inflammatory cytokines IL-1β, IL-6, and IL-8. ST6GAL1 knockdown alone led to a significant increase in mRNA levels of IL-1β, IL-6, and IL-8 (**Figure 3A**). Overexpression demonstrated upregulation in IL-8 but changes were not significant for IL-1β or IL-6 (**Figure 3A**). Interestingly, IL-6 protein secretion was higher in ST6GAL1 knockdown cells (**Figure 3B**) compared to the control and ST6GAL1 overexpressing cells, corroborating the changes in mRNA expression. In contrast, IL-8 secretion was not statistically different in the supernatants of the groups (**Figure 3C**), while IL-1β was below the limit of detectability (data not shown). These data suggest that loss of ST6GAL1 leads to increased IL-6 protein levels in HBECs that is not observed with IL-8.

### IL-6 Expression and Secretion Is Increased by Cigarette Smoke Exposure in HBECs and Partially Rescued by ST6GAL1 Overexpression

**Figure 4:**
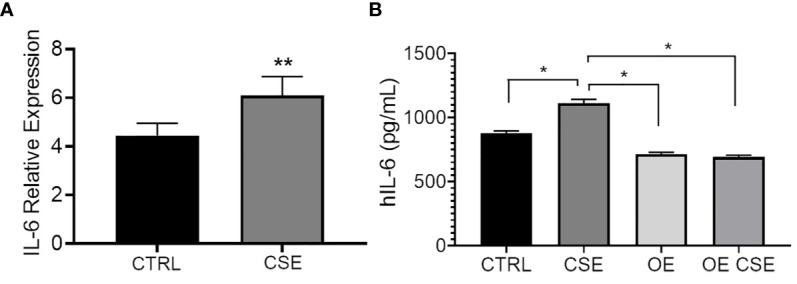
IL-6 expression and secretion is increased following cigarette smoke exposure and attenuated by ST6GAL1 overexpression in HBECs. **(A)** Relative mRNA transcript levels of IL-6 in control (CTRL) and CSE treated HBECs. **(B)** IL-6 protein levels from supernatants of control, and ST6GAL1 OE HBECs exposed to CSE for 24 hours. CTRL, control; CSE, cigarette smoke extract/medium; and OE, ST6GAL1 overexpression. All experiments were reproduced 3 times and done in triplicates with bar graphs indicating means ± SEM with *p < 0.05 and **p < 0.01.

To determine whether CSE affects IL-6 expression and secretion similarly to ST6GAL1 knockdown, we subjected HBECs to CSE for 24 hours. mRNA upregulation (**Figure 4A**) and protein secretion of IL-6 (**Figure 4B**) were observed following CSE exposure in HBECs compared to the controls and is consistent with our findings observed in the ST6GAL1 knockdown cultures (**Figures 3A, B**). When stable ST6GAL1 OE HBECs were exposed to CSE, there was no statistical change in IL-6 secretion levels when compared to the control group; however, the OE cultures showed a statistically significant reduction in IL-6 secretion compared to CSE alone (in the control group) (**Figure 4B**). Altogether, these findings suggest a potential link between the loss of ST6GAL1 and CSE induced IL-6 production in HBECs that is partially blocked by ST6GAL1 overexpression.

### Inhibition of ST6GAL1 Secretion Reduces Sialylation in HBECs Similar to CSE

**Figure 5:**
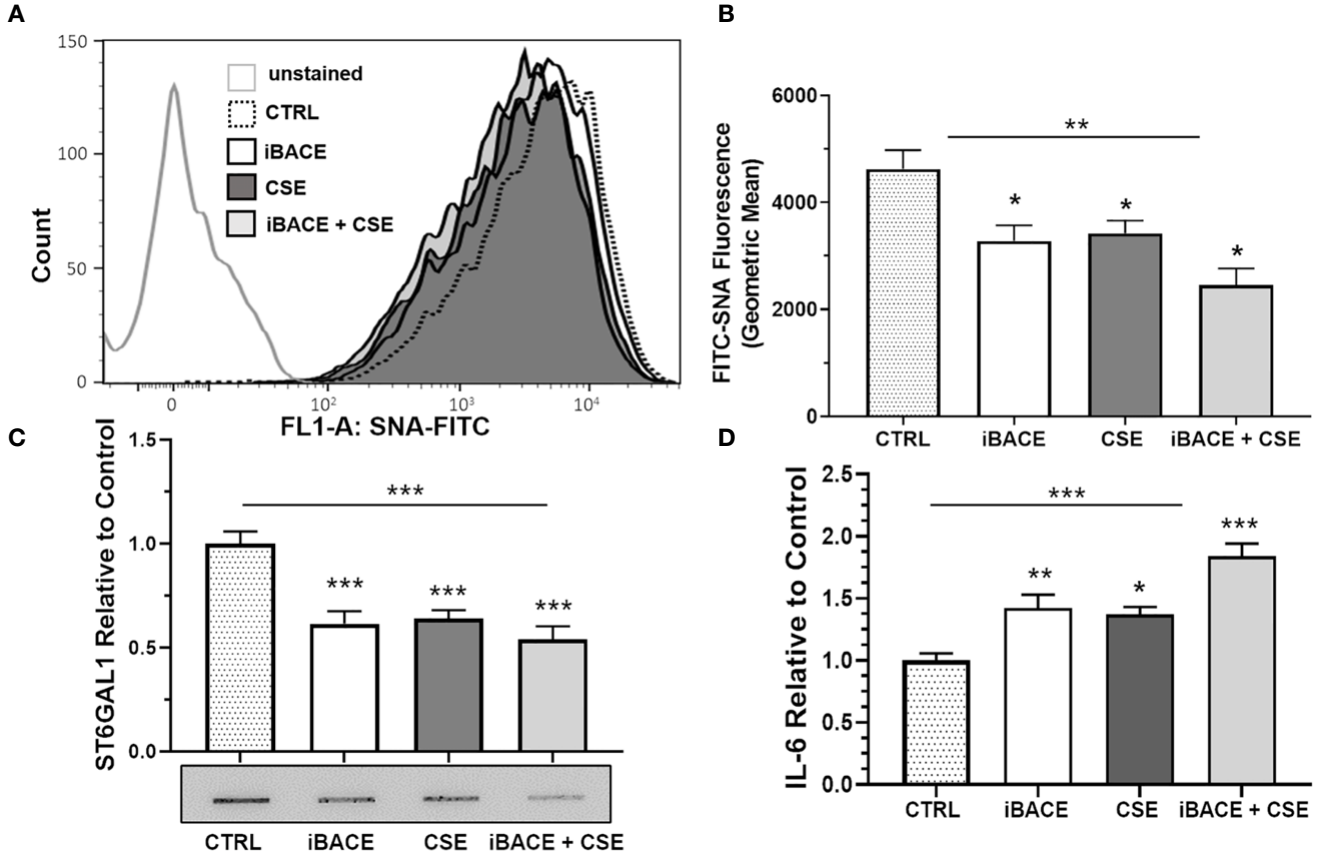
Inhibition of ST6GAL1 secretion reduces α2-6 sialylation and increases IL-6 secretion similar to CSE. **(A)** Flow cytometric analysis of α2-6 sialylation using SNA-FITC labeling of HBECs (CTRL, ST6GAL1 KD, and ST6GAL1 OE) following treatment with and without iBACE and CSE for 72 hours. **(B)** 10,000 events were collected for each group, analyzed, and shown as the geometric mean. **(C)** Densitometry and a representative slot blot of secreted ST6GAL1 collected from conditioned medium following incubation with iBACE, CSE, or both. As a loading control, the supernatants were normalized to total cellular protein. **(D)** IL-6 secretion was determined from conditioned medium using an ELISA kit to the IL-6 ligand following same treatments as **(C)**. Experiments were performed in triplicate using three separate experiments. SNA, Sambucus Nigra Lectin; CTRL, control; iBACE, beta-site amyloid precursor protein cleaving enzyme 1 inhibitor; and CSE, cigarette smoke extract. All bar graphs are means ± SEM with *p < 0.05, **p < 0.01 and ***p < 0.001.

Previous reports have shown that BACE1 (beta-site amyloid precursor protein cleaving enzyme 1) cleaves and releases ST6GAL1 from the trans-Golgi into the secretory pathway (41, 42). Therefore, we wanted to determine whether BACE1-dependent ST6GAL1 cleavage and secretion was necessary for any of the α2-6 sialylation in HBECs. Both the expression of BACE1 and ST6GAL1 has been shown previously in the lung epithelium (20, 43, 44). BACE1 inhibition in the HBECs resulted in a significant decrease in α2-6 sialylation levels when compared to controls and similar to the level of reduction when compared to CSE (**Figures 5A, B**). Combined, BACE1 inhibition and CSE resulted in more of a reduction in α2-6 sialylation (**Figures 5A, B**). As expected, ST6GAL1 secretion into the HBEC culture medium was reduced by BACE1 inhibition (**Figure 5C**), which was determined by slot blot and densitometry analysis. Interestingly, ST6GAL1 secretion was reduced following CSE and with both iBACE and CSE (**Figure 5C**). These data suggest that cigarette smoke extract exposure or blocking ST6GAL1 cleavage by BACE1 inhibition partially reduces secretion of ST6GAL1 and levels of α2-6 sialylation in HBEC cultures.

### BACE1 Inhibition Increases IL-6 Secretion

To determine the effect of BACE1 inhibition on IL-6 secretion, we subjected cells to the same treatments as shown in **Figure 5C** and measured IL-6 levels in culture medium by ELISA. IL-6 secretion from HBECs was increased following BACE inhibition, CSE administration, or both (**Figure 5D**). These findings suggest that blocking ST6GAL1 cleavage and secretion by BACE1 inhibition leads to an increase in IL-6 secretion from HBECs similar to *in vitro* cigarette smoke extract exposure (**Figure 4**).

### Circulating ST6GAL1 Levels Are Lower in COPD Patients and Associate With Worse Clinical Outcomes

**Figure 6:**
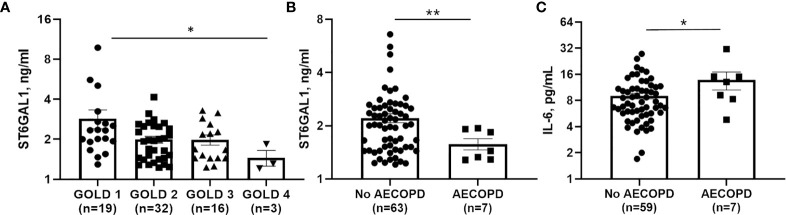
Circulating ST6GAL1 levels are lower in COPD patients and correlate with poor outcomes. **(A)** GOLD group status 1-4 and associated levels of ST6GAL1 in a COPD patient cohort. Circulating levels of ST6GAL1 **(B)** and IL-6 **(C)** and their respective association with acute exacerbations in COPD. Patients were classified based on severity of the COPD using the Global Initiative for Chronic Obstructive Lung Disease (GOLD) guidelines (30846476), which are as follows: GOLD 1: Mild (FEV1 >= 80% predicted); GOLD 2: Mod (FEV1 between 50 and 79% predicted); GOLD 3: Severe (FEV1 between 30 and 49% predicted); and GOLD 4: Very severe (FEV1 <30% predicted). Shown are the mean ± SEM, where n = subjects per group. *p < 0.05 and **p < 0.01.

To determine a potential clinical impact for ST6GAL1 and IL-6, we analyzed plasma levels in 70 COPD subjects, with characteristics displayed in **Table 1**. Median [IQR] circulating ST6GAL1 levels were 1.93 [1.44-2.48] ng/ml, while the mean ± SEM was 2.26 ± 1.33 ng/ml for the cohort. Circulating ST6GAL1 levels positively and significantly correlated with post-BD FEV1 percent predicted (Pearson’s correlation coefficient r=0.36, p=0.003), post-BD FVC percent predicted (r=0.30, p=0.011), and were inversely associated with GOLD stages (**Figure 6A**). However, ST6GAL1 was not correlated with dyspnea as measured by MMRC (r=0.19, p=0.10) or other respiratory symptoms measured by BCSS (r=0.15, p=0.22). Seven individuals (9.7%) experienced an AECOPD during the 1-year of follow-up. Circulating ST6GAL1 levels were lower among these individuals that experienced an AECOPD compared to the group that was AECOPD-free (**Figure 6B**; Median [IQR]: 1.44 [1.29-1.91] ng/ml *vs* 2.02 [1.48-2.58] ng/ml, p<0.001). In a logistic regression model adjusting for post-BD FEV1 percent predicted and smoking status, circulating ST6GAL1 levels were associated with decreased odds for AECOPD, though this failed to meet statistical significance (OR 0.14, 95%CI 0.02-1.19, p=0.072). Not unexpectedly, plasma IL-6 levels were higher among the AECOPD group compared to the non-AECOPD group (**Figure 6C**; Median [IQR]: 13.1 [8.30-15.1] ng/ml *vs* 7.44 [5.70-11.0] ng/ml, p=0.035). Circulating levels of ST6GAL1 and IL-6 were not associated (r=0.08, p=0.50). These translational findings support our *in vitro* data demonstrating loss of ST6GAL1 and increased secretion of IL-6, which may be linked to prognostic clinical outcomes in COPD patients.

**Table 1:** Baseline characteristics.

	COPD (n=70)
Age, years	59 ± 9
Male sex	36 (51%)
White race	34 (47%)
Smoking Status Current Former Never	34 (49%) 36 (50%) 2 (3%)
Pack-year history of smoking	36 ± 25
Post-BD FEV1, pct predicted	65 ± 22
Post-BD FVC, pct predicted	84 ± 16
FEV1/FVC	0.59 ± 0.16
MMRC score	1.8 ± 1.2
BCSS score	4.3 ± 2.7
Median [IQR] plasma ST6GAL1	1.93 [1.44-2.48] ng/ml

Data expressed as mean ± S.E.M. or n (%). BD, bronchodilator; FEV1, forced expiratory volume in 1-second; FVC, forced vital capacity; MMRC, modified medical research council; BCSS, breathlessness, cough, and sputum scale.

## Discussion

Our study is the first to link reduced circulating ST6GAL1 levels and increased IL-6 levels with acute exacerbations in COPD patients. These findings are complemented by our *in vitro* findings indicating that loss of ST6GAL1 results in decreased α2-6 sialylation and increased secretion of IL-6 in HBEC cultures under basal conditions. Our results also showed that exposure to cigarette smoke or BACE1 inhibition resulted in decreased ST6GAL1 secretion and loss of α2-6 sialylation. Conversely, CSE or BACE1 inhibition increased IL-6 expression/secretion consistent with the loss of ST6GAL1. Altogether, these results suggest that loss of ST6GAL1 function, through knockdown or blocking its proteolysis, augments IL-6 secretion in HBECs and is associated with poor clinical outcomes in COPD.

Recently, ST6GAL1 was recognized for its role in asthma (20). In this report, Zhou and colleagues showed that ST6GAL1 regulated airway epithelial cell differentiation and type-2 inflammation in asthma through altered mucin glycosylation and cell proliferation. The role of ST6GAL1 has also been shown in other diseases and their associated complications. For example, increased cigarette smoke exposure was shown to alter sialylation of the fallopian tubes potentially through ST6GAL1 in ectopic pregnancy (40). In addition, changes in protein sialylation were shown to contribute to fatty liver deposition and various inflammatory responses (45). Our findings demonstrate that loss of ST6GAL1 or exposure to cigarette smoke leads to decreased sialylation (**Figures 1**, **2**) and increased IL-6 expression/secretion in bronchial epithelial cells (**Figures 3**, **4**). These findings together highlight the potential importance of ST6GAL1 and α2-6 sialylation in inflammation and other cellular processes in the lung as well as other tissues that may be impacted by inflammatory mediators such as cigarette smoke.

Several reports have shown that ST6GAL1, a normally trans-Golgi network resident protein, can be cleaved from its membrane bound form to a soluble/secreted protein by BACE1 (41, 42, 46). BACE1 is the same enzyme identified for cleavage of the amyloid precursor protein involved in the pathogenesis of Alzheimer’s disease (47–50). Interestingly, our knowledge of the BACE1/ST6GAL1 interaction is still limited; however, BACE1 has been shown to be responsible for release of ST6GAL1 into the blood (51). BACE1 expression has been shown to affect the sialylation of soluble/cell surface glycoproteins through cleavage of ST6GAL1 (52) suggesting that the soluble form of the ST6GAL1 still has activity when released [others have shown that ST6GAL1 has activity in a secreted/soluble form (53)]. Additionally, secreted/circulating ST6GAL1 has been associated with inflammation (45, 54, 55). Here, we show that inhibition of BACE1 reduced ST6GAL1 secretion and α2-6 sialylation and resulted in augmented IL-6 secretion in HBECs similar to that found with CSE treatment (**Figure 5**).

In a previous report, Nasirikenari and colleagues demonstrated that administration of ST6GAL1 reduced infection in a mouse model of acute lung inflammation, while transient depression of circulating ST6GAL1 accompanied acute airway inflammation (56). In parallel experiments, they showed that inflammatory cytokine release was suppressed by recombinant ST6GAL1 infusion in these mice and suggested a potential role for ST6GAL1 in diseases like COPD. In a small COPD patient cohort, we observed an inverse association between circulating ST6GAL1 levels and lung function and GOLD stages (**Figure 6A**), suggesting that ST6GAL1 may have a protective role in maintaining lung function, potentially through anti-inflammatory mechanisms. We also found that ST6GAL1 levels were lower among the group that experienced an acute exacerbation compared to those that did not (**Figure 6B**) further supporting the hypothesis that ST6GAL1 may have a protective effect in COPD. Since our cohort was small, more studies are needed in a larger patient population to validate of these findings and to determine the use of ST6GAL1 as a potential prognostic marker of disease symptom severity.

In this same patient cohort, increased plasma IL-6 levels were shown to positively associate with acute COPD exacerbations (**Figure 6C**). IL-6 has been shown to be upregulated in COPD exacerbations (24) and was also suppressed/enhanced by the increase/reduction of ST6GAL1 in acute lung injury mouse models studied by Nasirikenari et al. (56). These findings are consistent with our *in vitro* data showing loss of ST6GAL1 led to increased IL-6 expression/secretion (**Figure 3**). Moreover, BACE1 inhibition (reduction in ST6GAL1 cleavage/secretion and α2-6 sialylation) or cigarette smoke exposure resulted in similar increases in IL-6 (**Figure 5**).

Our study is not without limitations. Our translational results were generated from a single center study and contained a relatively small sample size. However, this small sample population was offset by a well-characterized dataset and complete follow-up. In addition, ST6GAL1 was measured from plasma of these patients and not from lung tissue or sputum, which was not practical for this study. Analysis in these samples would have been a more ideal and direct measure. Still, these findings in plasma were robust and improved the overall generalizability to other human cohorts. Finally, studies here were performed with HBECs and we cannot rule out the fact that circulating ST6GAL1 may be coming from other tissues (e.g., liver). In future studies, we plan to utilize primary cells and tissue obtained from COPD subject studies to obtain a more direct measure.

Altogether, our results along with the previous literature suggest that ST6GAL1 potentially has an important role regulating the inflammatory cytokine response in patients diagnosed with COPD. Further, although future studies are needed, our work suggests that circulating ST6GAL1 levels might serve as a potential therapeutic marker of acute COPD exacerbation and of inflammatory lung disease progression.

## Data Availability Statement

The original contributions presented in the study are included in the article/supplementary material. Further inquiries can be directed to the corresponding author.

## Ethics Statement

All protocols were approved by the Institutional Review Board of the University of Alabama at Birmingham. The patients/participants provided their written informed consent to participate in this study.

## Author Contributions 

SK, JB, and JW contributed to the concept and/or design of the study. SH, ME, RD, RZ, SB, PC, SV, EH, JW, JB, and SK contributed to the acquisition of the data and SH, JW, SK, and JB contributed to the analysis and interpretation. SK, SH, JW, and JB drafted the manuscript. All authors contributed to the article and approved the submitted version.

## Funding

This work was supported by the Flight Attendant Medical Research Institute (YFAC152003 to S.K.), the Cystic Fibrosis Foundation (CFF P30 DK072482 and CFF Rowe19RO to S. K.) and the National Institutes of Health (R00HL131866 and R01HL152246 to JB; R03AG059994 to SK; and R01HL148215 and R35HL135710 to JW).

## Conflict of Interest

JW receives grant support from Bayer AG, Vertex Pharmaceuticals, Grifols, Verona Pharma, Mereo BioPharma, and Arcus-MED to conduct research independent from the work in the current manuscript and has been a consultant to AstraZeneca, Takeda, and GlaxoSmithKline for projects unrelated to the content of this work.

The remaining authors declare that the research was conducted in the absence of any commercial or financial relationships that could be construed as a potential conflict of interest.
